# Data roles: youth mental health outcome measures and the young people who defy them

**DOI:** 10.1057/s41292-025-00366-1

**Published:** 2025-10-18

**Authors:** Rosie Jones McVey

**Affiliations:** Centre for Cultures and Environments of Health, University of Exeter, Queens Drive, Exeter, EX4 4QH UK

**Keywords:** Data, Outcome measures, Youth, Mental health, Wellbeing, Accountability

## Abstract

Health measurement shapes peoples’ political relationships with the state, with services, with one another, and with oneself. But what are the political dynamics at play when people can’t/won’t/don’t have health measurements taken? And what is the political predicament of those whose needs, values, and experiences don’t fit within the measures available? This paper presents a case study of one youth mental health service’s efforts to improve their collection of outcome measures, and reinvigorates the concept of ‘sick role’ to describe young people as defying the ‘data roles’ expected of them. The concept of data roles draws attention to the political dynamics of measurement on two interlinked scales: the interpersonal, embodied measurement encounter; and the systemic care-measurement assemblage. In the case reported here, measures are hard to collect given the ‘routinized intimacy’ required, and the restrictive, normative, individualised understandings of need inscribed within available measures. Yet defying measurement equates to a marginalised, precarious political position for young people and for the services that support them. In sum, the data roles expected of young people ask too much of them, and do too little for them.

## Introduction

In 2024, in the UK, in a large conference room, around 100 youth mental health service leaders and staff members gathered at a networking and professional development event. Each table full of service providers was asked to write their most pressing concerns on Postit notes and put them onto a shared board. Amongst the constellation of concepts raised through that meeting, one requirement shone particularly clearly, since almost every Postit contained a version of it: “help with collecting outcome data”.

While outcome measurement was required for bolstering referral and funding pathways, many of the young people these services supported simply didn’t seem to fit within any of the measurement tools that carried legitimacy with state agencies or substantial funding bodies. Many service users either didn’t measure well (wouldn’t fill in the required forms consistently, completely, authentically), or wouldn’t be measured at all. Beyond this one professional development event, I came to learn that these problems were faced by a broad range of charity-based and state-based mental health and youth support services (more on which shortly).

In fact, the attendants of that event did not run *just any* youth mental health services, they ran equine-assisted services—which use horses to help with therapy or education (Jones McVey 2025a; Malcom et al. 2018; Tomlinson 2024) However, the only relevant aspects of that modality for this article are that it was an emerging and growing sphere of practice, still relatively unregulated, and usually delivered by charities. Therefore, like other alternative and innovative approaches to supporting youth mental health, equine-assisted services held new promise but somewhat fragile legitimacy amid the fierce competition that existed for insufficient mental health funding from the state^1^ or other avenues. While most equine-assisted services aspired towards state commissioning arrangements, with the hope that these would bring consistency and legitimacy, in the meantime, many relied upon public donations and private trusts. A large portion of their workload was fundraising, and while some equine-assisted services were thriving, many others could barely make ends meet, despite substantial demand for their programmes.

Thus far, more academic attention has been paid towards the workings of power when measurements *are* taken, audits *are* completed, or data *is* produced, than attention has been paid towards what is happening when measures end up missing. Such studies have attended to measurement and health (Bivins 2020; Lupton 2016; Rose 1990); studies of audit culture (Lambert 2006; Power 1997; Strathern 2000; Zacka 2017), and studies of the datafication or ‘enumeration’ of morally valued goods (Hacking 1982; Sangaramoorthy and Benton 2012), such as global health (Adams 2013, 2016; 2017) public health (Hoeyer et al. 2019; Saluk 2022), development (Taylor and Broeders 2015), and education (Jarke and Breiter 2019). This growing array of studies has substantive breadth and depth beyond the scope of this article to overview. Yet amongst this variety is a common critical observation that inequalities in power, wealth, and health can be perpetuated by measurement procedures, by dint of the political distinction between those who are counted and those who do (or orchestrate) the counting. Often drawing on Foucauldian heritage, such studies also show that power works in ways that are not immediately apparent—at least, not to those who are being measured, or self-measuring. A classic and very relevant example is Nikolas Rose’s description of the way new technologies for measuring children’s behaviours and developments led to the formalisation of developmental norms, and the subsequent anxious appeals of mothers to experts for guidance to keep things on track (1990). Thus, families became self-monitoring and self-managing in line with state ideas: they developed ‘governmentality’. More recent examples describe people as subjects of ‘dataveillance’ (Van Dijk 2014) and demonstrate how new digitised health-tracking technologies can invite people to inadvertently expose themselves to biopolitical control (Davis et al. 2023), corporate or managerial exploitation (Lupton 2016; Lupton and Williamson 2017), or loss of autonomy over their own ways of evaluating their lives and activities (Nguyen 2024).

Some studies have shown that people (or rather, some people) have more agency, or more complex forms of agency, in relation to their pursuit/non-pursuit of health measurement than might be expected (Kennedy 2018; Sharon and Zandbergen 2017). Consumers of health-tracking technologies may be seen as *actively choosing* to hand over some degree of agency to their tools, ‘opting out’ of having more independent control over health behaviours (Schüll 2016), or as demonstrating ‘soft resistance’ to metric design by each curating agentive and idiosyncratic relationships with health data (Nafus and Sherman 2014). In strong contrast with the case presented here, some young people and their families are keen to partake in the datafication of their behavioural diversities, Rayna Rapp (2016) explores the reasons and impacts of this volunteerism within new forms of brain mapping research). Other studies have shown that people can ignore, redeploy or ‘tinker with’ health quantifying technologies. For example, Anne Marie Mol demonstrated the idiosyncrasies with which people with diabetes did or didn’t complete their blood sugar measurements as directed (2008), while Amanda Lazar and colleagues (2015) showed that people can simply abandon health-tracking technologies that don’t align with their conceptions of themselves.

Despite these complex and nuanced forms of agency, one may be forgiven for thinking that choosing *not* to be measured indicates a position of relative empowerment, freedom, diversity, creativity, or autonomy. However, one of the most convincing and persistent aspects of critical accounts of health measurement is their observation of the unexpected and unintentional ways that power operates. Afterall, Foucault’s notion of power is a productive as well as restrictive one—in tracking their children’s development and health, the mothers that Rose described were both empowered as parents (in certain ways) *and* subscribed to state power (for a collection of more recent examples, see Sangaramoorthy and Benton 2012). This would suggest that being measured and not being measured are both complex political predicaments in which one’s measurements (or lack of measurements) hold possibilities for subjective interpersonal action *and* for systemic political impact, beyond individual invention or comprehension. Current ethnographic attention on users who withhold, obstruct or creatively engage with their health measurements tends to emphasise user agency, subjective experience, or idiosyncrasy, perhaps at the expense of investigating the unexpected systemic political predicaments that are brought about through individual deviance or disengagement from expected measurement practices.

In stark contrast are studies of public or global health that highlight what, or who, is left out in the collection of legitimised health data, including in the quantification of needs, or the creation of ‘reliable’ data sets. For example, Vincanne Adams has powerfully lamented the ‘erasure’ of contextualised and locally varied forms of health knowledge within the ‘tyrannical’ growth of evidence-based approaches to public and global health (2013, p. 58), and Elsa Fan and Elanah Uretsky have drawn together a special issue of anthropological contributions which highlight the exclusions and ‘blind spots’ of evidence and accountability measures in relation to health (2017). In these examples, if one defies measurement in one way or another (not necessarily intentionally, but by being outside/beyond the measure parameters), this may not equate to a position of creativity, flexibility or empowerment, but systemic neglect.

This perspective can find further support among those who have described data or measurements as part of an ‘assemblage’ (Ruckenstein and Schüll 2017, p. 270; Iliadis and Russo 2016; Hogle 2016). Discussions of ‘accountability assemblage’ (Hoeyer et al. 2019; Hogle 2019) and ‘data assemblage’ (Lupton 2016; Davis et al. 2023) (and I will use the term ‘care-measurement assemblage’) emphasise the political importance of the formats, mediums, and technologies through which measures are shaped, stored, and sorted. One growing interest in line with ‘assemblage’ thinking is the question of ‘data performativity’ (Blouin 2020; Kitchin 2014), which describes the way data can have something like agency in reshaping the understanding of problems it is set to investigate. For example, problems can become recognised (and therefore treated) in line with their most measurable attributes. Key questions then, are who, or what, are included or excluded from data sets, how ‘data gaps’ come about *beyond* the intentions of any individual humans or groups, and what sort of predicament follows not being counted.

Taken together, these critical aspects mean that when youth mental health service providers try, but do not manage, to get good mental health data from their service users, there is an interesting political predicament at bay: are service users escaping/defying normative measures and forms of control? And/or, are they marginalised within, or excluded from, definitions of ‘the problem’ and evaluations of ‘the solution’? And what sort of conceptual apparatus can help us draw attention to both of those parameters? This predicament of unmeasurability^2^ warrants further comparative study, of which, this article is one part.

The paper is centred on an ethnographic case study of one equine-assisted service’s efforts to collect better outcome data, though I also draw on broader ethnographic and interview data. The first section of the paper will orientate the case study within the political and ethical context of the datafication of UK youth mental health care, and finish with a description of the methods used. In the next section, I present the case study, demonstrating the challenges service providers faced in selecting an appropriate measure, and in collecting good quality data, under several sub-headings. During this section, I will assert the utility of studying ‘data roles’ within a ‘care-measurement assemblage’. Drawing on Talcott Parsons’ classic medical sociological term ‘sick role’ (1951), I will emphasise the socio-technical configuration of legitimacy. The final section is the discussion, in which I will develop the argument that the role currently expected of service users in measuring intervention outcomes is exclusionary, idealistic, and consolidates the misrecognition of marginalised young people’s needs. Yet, while acquiescing to the roles expected in measurement encounters is an unrealistic and unfair task, *not* completing outcome measures leaves both service users and service providers in precarious positions.

## Orientation

Like other examples of the ‘datafication’ of mental health in the UK (Pickersgill 2019; Bruun 2023; Armstrong 2016, 2023), *youth* mental health data have become increasingly morally and politically pertinent in the UK over the last three decades. Audit and evidence were key parts of New Labour’s modernisation of mental health services,^3^ and subsequent Coalition and Conservative policies have further insisted on the need to improve or even ‘revolutionise’ mental health data.^4^ Policies aim for more complete and reliable prevalence rates, treatment access rates, wait times, and treatment outcome data. But while the ‘datafication’ of health is often, rightly, understood by critics as caused by, and enabling, economised reasoning in health care (either in pursuit of state or donor spending efficiencies, or corporate profits, e.g. Adams 2016; Erikson 2012; Fan and Uretsky 2017), the story in relation to youth mental health data is morally complex.

The increasing importance of data in youth mental health care can be seen as part and parcel of an expanding legitimacy for diverse ways of understanding and treating mental distress. Faced with stark recognition of unmet needs, state agencies (e.g. from national services to commissioning groups to local authorities) have had cause to more concertedly explore alternative provisions that are less traditionally ‘clinical’, particularly those that are community-led, and charity run. This broadening aligns with the ‘person-centred’ approach of contemporary UK state health policy, which emphasises patient choice, diversity, and (at least ostensibly) the importance of supporting health within social context (e.g. NHS Long-Term Plan 2019). But also, this spread into new areas of the VCSE sector enables an increase in capacity through tapping into new resources (volunteer time, charitable donations, etc.). An expansion beyond biomedical, psychiatric approaches appears to go hand in hand with further outcome monitoring (Pickersgill 2019). Pickersgill charts how the adult IAPT programme (initiated 2008) was so influential in this respect that it changed the context for future healthcare developments (including for those described in this article). Rather than clinical qualifications (e.g. in psychiatry) earning legitimacy in treatment provision, the emphasis moved to services’ capacities to prove their worth with outcome data (for a similar pattern in global health, Fan and Uretsky 2017). This follows a trend associated with ‘managerialism’ in state health care and public services more generally—numerical audit becomes more important as part of the ‘arms distance’ broadening of public service delivery (Clarke and Newman 1997). The requirement on *charitable* services to prove their worth with outcome data resembles but differs from similar demands within NHS services (described by Pickersgill 2019; Armstrong 2023), in that those running ‘alternative’ services act as though they are on the cusp of measuring themselves in, or out, of significance to the state, rather than merely fulfilling its obligations.

Yet mental health data wasn’t only about new possibilities—and demands—for garnering the legitimacy of treatments. It was also about answering to the public, including young people themselves. Youth mental health has been publicly recognised as undergoing a growing ‘crisis’, both in terms of morbidity rates and inadequate treatment provisions, since well before Covid-19 lockdowns, though exasperated by those events (Merikangas et al. 2009; Samji et al. 2022). Within the context of this public crisis, data have been treated as something morally potent: a tool for demanding the better acknowledgement of neglected problems or peoples; even a medium for the witnessing of suffering and injustice. In policy documents, youth mental health data are rhetorically used to publicly acknowledge and define the extent of the crises, and improvements in data strategy are proposed in order to chart accountability for remediating the problem, at both government and service levels. While one might argue that such use of data is just statutory political posturing (and no doubt, that is as least partly apt), mental health data have also been the backbone of advocacy groups’ campaigns for better care, standing alongside young people’s testimony, and/or providing a format for young people’s testimony (e.g. Young Minds 2023) (on the tight/tensive relationships between testimony and data in mental health accountability procedures see Armstong 2023; Hoeyer and Langstrup 2021). Across many of the academic, political and professional service environments that I visited during research into equine-assisted therapies (2019–2024), there was a sense of hope and endeavour that better data would lead to support for *the right* treatments, and so better health justice.

That said, this seeming faith in data is not of a naïve sort: many of those working with youth mental health data were keenly aware of some of its challenges and weaknesses. For example, Department of Health efforts to transform children’s mental health care systems through the CYP IAPT programme(established in 2011) emphasised the need for better outcome data as one of its core remits. Yet the researchers responsible for designing the outcome data strategy and conducting the subsequent evaluation were carefully reflective about how to use the data collected—describing it under the acronym ‘FUPS’ for:

“flawed, due to missing or erroneously recorded data; uncertain, due to differences in how data items are rated or conceptualised; proximate, in that data items are a proxy for the focus of interest; and sparse, in that there may be a low volume of cases for key subgroups”. (Wolpert and Rutter 2018, p. 1)

Wolpert et al. recognise that FUPS youth mental health outcome data are not only a national problem, but an internationally persistent challenge (2016, also Fleming et al. 2016; Batty et al. 2013). Therefore, while ‘good quality’ outcome data may well hold moral, political and economic value, collecting ‘good’ outcome data clearly presented challenges, well beyond the realm of equine-assisted therapy.

Conversely, and importantly, the UK was also a political environment where youth mental health data could be disavowed or even ridiculed as new-fangled fuss. Youth mental health needs were seen by some as over-indulgence, and especially scoffed at when youth mental health data relied upon self-reported measures of health and wellbeing. For example, *Daily Mail* articles by Sarah Vine (2016) and Dr Martin Scurr (2024) mocked both mental health statistics, and young people themselves, as phony and unreliable.

Recognising the multifaceted moral, political and economic importance of data in this field and following Bruno Latour’s approach to engaged critique (2004), my aim is not to attempt to debunk youth mental health outcome data as a system that doesn’t, in the end, produce what Latour called ‘matters of fact’. Such an approach would devalue the concerted ‘caring-through-data’ (Kaziunas et al. 2017) that many of my interlocutors displayed, and risk fuelling the delegitimization of unmet needs or of economically vulnerable services. One of my interviewees—a research strategist who supported charities to improve their impact measures—beseeched me “you’re not going to portray us as neoliberal witches are you? We are trying to do good work here!” My aim instead is to approach the problem of measuring youth mental health with care, as a complex, multidimensional ‘matter of concern’ (Latour 2004), and to offer observations as a critical friend, with an ethnographic vantage point on the challenges of collecting not just ‘good’ mental health data, but mental health data that can do good.

## Method

The argument presented here draws on 14 months of ethnographic fieldwork (2020–2022), as part of a broader project investigating the moral and ethical dimensions of equine-assisted therapies for UK youth (2019–2024). During this fieldwork, I took on a participant observation role as a volunteer within three therapy centres. I also attended training and networking events for youth mental health provisions, I visited a further 8 equine-assisted service providers to get to know their programmes, and conducted in-depth, unstructured interviews with another 35 people who offered, or were in the process of setting up, equine-assisted services. The field of equine-assisted therapy includes a broad array of services, which differ in size and scope, and in terms of the qualifications of staff and the therapeutic or educational modality taken. All of those I spoke with reported challenges in collecting the outcome data that they required in order to sustain or grow their charities. In 2021, one of my key field sites, ‘The Stables’, was prompted to review and update their outcome measurement through recommendations from their funders and referrers (details removed for anonymity). The directors of The Stables asked if I would join the team of staff overseeing the outcome strategy update—herein the ‘Outcomes Team’—which consisted of one of the charity’s directors, two clinical psychologists, a youth worker, and the charity administrator. I used this as an opportunity to qualitatively study the challenges involved in updating the outcomes measurement procedures, though I collected ethnographic observations on these aspects in other settings also, including through focus group discussions (5, *n* = 14) and interviews with other stakeholders (*n* = 8) in the outcome measurement process, and I include some of those details here, using some fictionalisation to obscure the sources and employing pseudonyms to aid anonymity. The case study should be taken as indicative of the predicaments faced by many services, rather than an accurate (and therefore identifiable) portrayal of one. The safeguarding lead at The Stables has had the opportunity to read this draft and identify any concerns regarding identifiability or unintended harms.

## Case study

### Logical measures

Before the Outcomes Team was established, The Stables were already using evaluation forms that they had written themselves. These involved a mixture of tick box scales for service satisfaction and short answer questions, with a slightly different version for young people and for their teachers, parents or carers. But following the recommendations of a commissioning body, the plan was to add a measure that could detect *outcomes* (rather than experiences) in a more rigorous and consistent manner, enabling easier commissioning comparisons between different services, or comparisons about outcomes for different groups within similar services.

The Stables were keen to use a ‘patient reported outcome measure’ or ‘PROM’—this means that young people themselves, rather than mental health staff, teachers, or parents, would be the ones to report on the state of their health or wellbeing at the start and end of the programme (note this ‘inclusion’ of voice is not the same as a *user-generated* outcome measure Rose 2011, or forms of user-designed care that sit outside of outcome measures, Adame and Knudson 2007). PROMS are gaining currency in youth mental health care (Fleming et al. 2016). Given a broader context in which the power dynamics between service users and service providers have been much critiqued, the notion of young peoples’, or service user’s, voices, carries moral, political and clinical import (Jones McVey 2023, 2025b, which can be misappropriated, e.g. Gorman and LeFrançois 2017, p. 110). For The Stables, PROMS enabled what felt like more equitable and appropriate forms of measurement than clinician-reported or carer-reported measures, particularly because staff often found themselves disagreeing with the negative portrayals that could be reported by teachers, previous therapists or parents during referral, and were also cautious about the suitability of their own perspectives as authorities on what ought to count as progress. However, it will become clear as we move through the case study that power dynamics are reconfigured, rather than completely remediated, when young people are enabled/required to complete their own health and wellbeing assessments.

One of the clinical psychologists on The Stables’ Outcomes Team, Peter, had recently completed some training through CAMHS in improving outcome measurement. Peter’s training had introduced him to the Child Outcomes Research Consortium, or CORC. CORC was established in 2002, supported by TheAnna Freud Centre (daughter of Sigmund), to bring together researchers and practitioners of children’s mental health care, in order to work towards better ways of understanding and evidencing outcomes. CORC provided a free online ‘evidence-based practice logic model’ which contained four boxes for services to fill in.

The first box described the ‘Target’—the people the service was for. The second described the ‘Intervention’—what it was that the service offers. The next box was for ‘Change Mechanisms’—how it was that the intervention was expected to change anything. And finally, the ‘Outcomes’—visible through comparison of the same measurement at two (or more) time points. However, it quickly became clear this was going to become an unwieldy document, requiring reems of tiny font in order to fit into the page and incorporate the detail necessary. This is because the ‘Target’ population was very broad—sharing only age as a common denominator. They included, refugee children who visited The Stables with an intermediary charity; school groups from Pupil Referral Units (who were permanently excluded from mainstream school); children with experience of the care system as part of a package of Local Authority funding; CAMHS individual referrals; parental referrals; and mainstream school referrals. The challenges these young people faced were sometimes described by referrers as (a non-exhaustive list): behavioural challenges at school and/or home; trauma recovery; anxiety; school refusal; selective mutism; self-harm; suicidal ideation; social skill or personal development for young people with autism or other neurodiversities; eating disorders, OCD; conduct disorders, impulse control challenges, and/or depression. The Stables’ staff didn’t always agree with the accuracy or utility of these categorisations, and some people had suspected rather than diagnosed conditions (either because of very long wait times, or resistance from families/young people against diagnoses).

Even within each of the above categories, the way The Stables worked was too personally variable between service users to be amenable to the logic model. One young person with suspected depression was able to practice motivating a sluggish horse and then used those embodied techniques (breathing exercises, postural adjustments) to motivate his own daily activities at home. Another young person was able to talk about her “depressed feelings” (her term) with a support worker while walking through the countryside lanes with a horse for company; while a third described as “depressive” in her referral found unparalleled comfort in the physical sensation of stroking the horse while she resisted any attempt at 'therapy-talk'.

It became clear that it was unviable to measure different change mechanisms for different young people (not to mention statistically unwise, in shrinking the population size of each measure). Pragmatically, it was not only an issue of staff managing multiple measures for different individuals at the same time, it was also that part of the programme ethos was to figure things out with young people, flexibly, intuitively, experimentally, and reflexively as the programme progressed. Nobody quite knew what young people needed until during or after the event, so ‘change mechanisms’ were very difficult to pre-determine. The only option for The Outcomes Team was to generalise up to something so broad that (a) the diverse positive impacts for as many young people as possible would be included and (b) for each young person, unexpected shifts in understanding what the intervention was for, or how it might work best, would still fall under the net caught by the data strategy. With this in mind, The Outcomes Team landed on testing either for ‘wellbeing’ or ‘resilience’. One significant downside to generalising up to these broad measures was that profound, personally meaningful positive impacts could barely show up on the measure (Nguyen 2024; Adame and Knudson 2007). A young man speaking the first words uttered outside of his home in 2 years; another attending whole sessions against a backdrop of persistent disengagement; a young woman feeling safe enough to make an important disclosure—none of these ‘showed up’ as statistically strong improvements in wellbeing or resilience.

### Individualising and idealising health

The terms wellbeing and resilience resonated well with the moral ethos of The Stables, because they extended beyond diagnostic criteria or measures of function. As with other ‘alternative’ mental health provisions, many staff at The Stables were critical or at least cautious of more conventional biomedical or psychiatric approaches to mental health and of more conventional pedagogical approaches to adjusting young people’s behaviour, too (resonating with critical approaches towards education, e.g. Varenne and McDermott (2018), and mental health treatment, e.g. Recovery in the Bin, Edwards, Burgess and Thomas 2019). One member of the youth support team at The Stables, Stacey, told me that following her own “mental health journey” (her term), she had come to realise that nothing was wrong with these young people at all, it was the schooling system, the class system, and the race system that was “gone to shit”. Not all held such strongly worded views as Stacey, and several staff and young people valued certain sorts of medical mental health diagnoses, or collaborated with some aspects of biomedical care. However, while there were a range of critical attitudes towards various medical approaches, *all* could congregate behind the idea that what The Stables did well was to help people cope in an unfair world (aka resilience), and/or, to reduce their suffering and improve their flourishing (aka wellbeing) as best as possible despite the challenging world they lived within.

With this ethos and modality of work in mind, the Outcomes Team hit the first of many ‘impossible situations’ (Zacka 2017, more on this phrase to follow). Some measurement tools seemed to do better at fitting with the ethos of contextualising health, through asking questions about young people’s environments within their measures of resilience or wellbeing. For example, the Child and Youth Resilience Measure (CYRM) asks respondents to rate, from 1 to 5, how much they agree with the statement "If I am hungry, there is enough to eat", as well as "I am treated fairly in my community". However, it was unstrategic to consider a measure that included criteria that The Stables had little chance of influencing. In the end then, the Outcomes Team moved towards wellbeing and resilience scales that took more of an individualised approach. However, it then became challenging to avoid scales that idealised certain character traits or portrayed subjective states as social skills or obligations. For example, the Stirling Wellbeing Scale asks respondents to rate "I have always told the truth".

The handling of truths may be a complex, slowly shifting, and nuanced affair for those with experience, or fear, of the criminal justice system; for those with experience, or fear, of the care system with concerns about exposing parental abuse or neglect; for those who are victims of crimes they do not want to report; for those undergoing immigration proceedings; for those with distressing or intrusive thoughts or psychological symptoms that they do not want to expose; and so on. This means the top-scoring ideal of *always* truth telling might not only be unrealistic (and, ironically, liable to misreporting) but also a poor measure of a persons’ capacity to navigate ‘truth’ within complex environments in such a way that really would be conducive to positive wellbeing. More broadly speaking, The Outcomes Team could find no measures that were sensitive to the changes made by an individualised intervention (i.e. dealing with individuals one at a time, via referral routes) on a generalised scale (i.e. measuring those with depressive symptoms alongside those who ‘can’t sit still’ and more), without inappropriately idealising the sort of individual that interventions were supposed to be creating and subsequently giving a sense of negative judgement or deficiency for those who don’t measure well.

This idealisation caused problems in terms of measure coherence (did the measure capture the sort of support The Stables offered?) but also for measure amenability (would these idealised measures be tolerated or rejected by respondents?) Several available scales of wellbeing read like a list of Polly-Anna-ish attributes, including items like "I’ve been feeling cheerful about things" "I like everybody I have met" and even "I always share my sweets". These items seemed so disjointed from some service users’ life experience that The Outcomes Team were concerned they risked negatively impacting upon relationships between staff and service users. This was a particular concern given, as described above, the importance of slowly evolving, flexible, varied, and responsive relationships as a key factor in the treatment modality itself.

### Care-measurement assemblage

The disjuncture between the available measures and the experienced reality can be seen as a matter of epistemic injustice (Fricker 2007) since it renders young people unable to express their predicaments within the confines of the legitimised heuristics available—they have to fit into boxes they didn’t design (Rose et al. 2011). But this epistemic injustice is produced not only by human-held prejudices, but by a complex socio-technical system. In part, it is an attribute of the bias within psychological research towards particular cohorts. This bias is not easily overcome because it reflects the relative ease of accessing different sorts of cohorts for procedures that ‘validate’ psychometric measures. Validation of psychometric tools for adolescent populations usually use school settings, and invite focus group feedback from a smaller cohort—but those not at school, not taking part in the validation exercise even if at school, and/or, unlikely to join focus groups, are perhaps more representative of the Stables’ cohorts. The sense of judginess and responsibilization that some measures could convey was worsened by the format in which they were used as ‘before’ and ‘after’ measures of intervention success. Questionnaire items (e.g. "I have been feeling optimistic about the future") may not have been interpreted as judgemental if distributed in a more neutral context, say, if the questionnaire arrived to broad swathes of the population by post. Some questionnaire items may be even argued to represent common goods (i.e. perhaps everybody would like to feel optimistic). Nonetheless, these items could come to appear to identify what needs fixing or changing when rated as part of receiving a service, as though one *ought* to be feeling more optimistic, inadvertently redeploying a common obligation on those suffering with mental health needs to be ‘cured’ rather than understood, accommodated, and supported (Clare 2017).

**Fig. 1:**
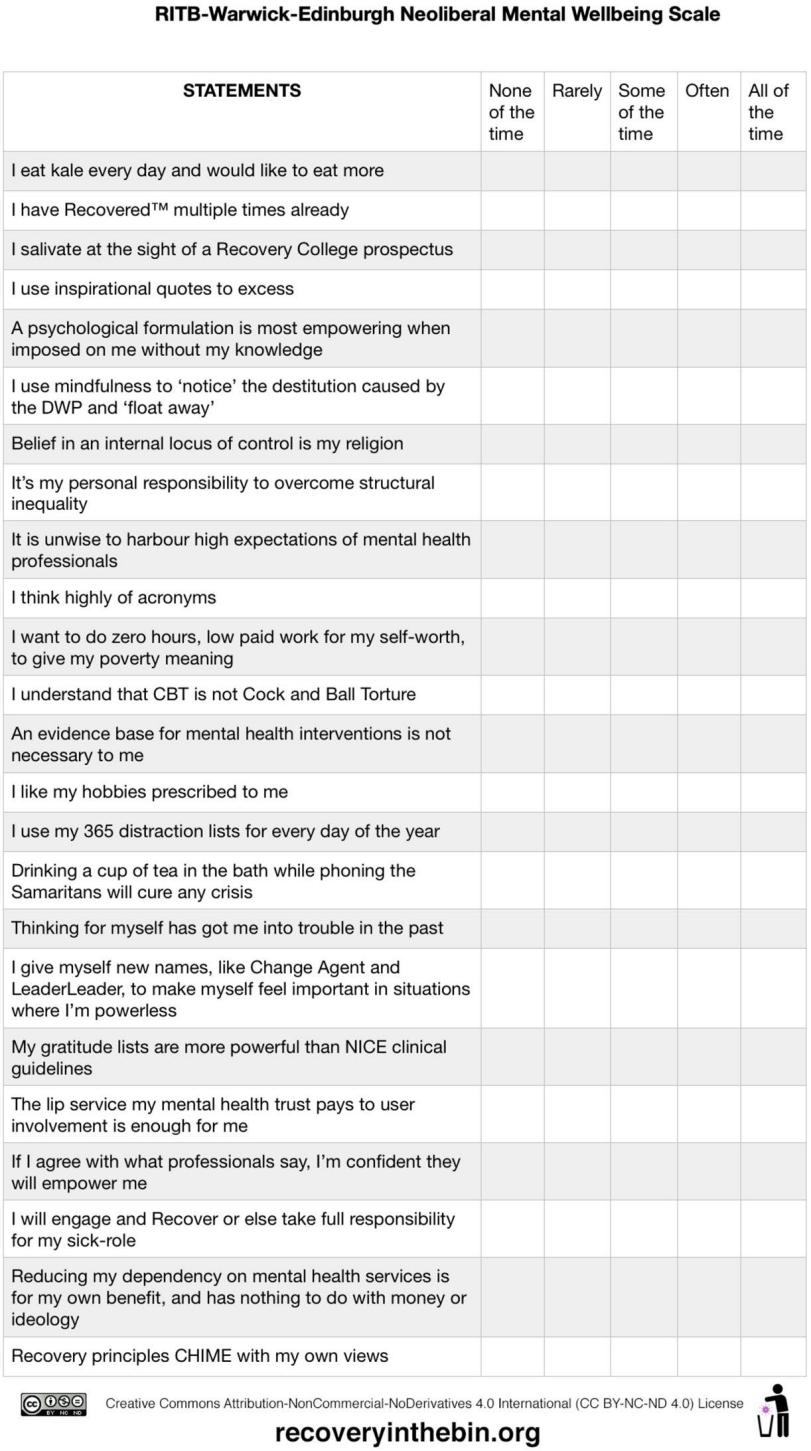
RITB Warwick Edinburgh Neoliberal Mental Wellbeing Scale (2019)

The irony and judgement inherent in such a measurement system has attracted the critical attention of survivor-led groups and advocates, such as ‘Recovery in the Bin’ (2019) who have created a satirical version of the WEMWBS, which opens with the item “I always eat kale” (Fig. 1). Recovery In The Bin criticize outcome measurements as examples of ‘NeoRecovery’ (neoliberal appropriation of Recovery movements) which, they argue, ultimately serve a capitalist agenda to get people back into the workforce and costing the state as little as possible (Recovery in the Bin, Edwards, Burgess and Thomas 2019).

Yet the term ‘assemblage’ is helpful here for highlighting that such responsibilization is exasperated through a broad, intricate, and dynamic socio-material system, as opposed to merely pursued by certain human actors (like mental health staff or politicians), or inscribed within cultural norms (like capitalism), or saturating certain biassed measures (like wellbeing scales). While the term assemblage already denotes “arrangements of practices, technologies and theories that configure action in sociotechnical space” (Hogle 2019, p. 558), I find the term ‘care-measurement assemblage’ helpful, for critically emphasising instances where systems of care provision rely upon and are shaped by systems of measurement (and vice versa). The hyphen can separate as much as it holds together (van Baalen 2023), as such, the term invites critical enquiry about the proper relationship between those two terms, as much as observation of any particular empirically observable relationship.

At the Stables, the care-measurement assemblage organises flows of money, data, knowledge and people. Organising factors include the individualisation of referral systems; the availability of certain cohorts to psychometric scale development; the validating power of user-reported measures alongside the potential leverage of scalable, quantifiable data; the broadening of forms of therapeutic expertise alongside the need to compete for resources on the basis of change made per person per time unit. This assemblage is shaped by progressive incentives as well as economising logics and has as much to do with the shifting affordances of data as it has to do with cultural and political dynamics.

### Routinized intimacy

While the Outcomes Team didn’t want to ask young people questions that may make them feel unfavourably judged in line with inappropriate ideals (as in, "I always share my sweets"), they were also advised by a research strategist, Dana, not to shy too quickly away from asking *any* questions that generated low starting scores from respondents. Dana explained that questions that generated low scores might better capture the depth and extent of young people’s problems, such as to give good room for improvement in scores through the intervention, and also to bolster calls for the need for further funding given the state of distress. In her experience, one of the problems charities faced was young people who were thought to have significant needs measuring too well at the start of interventions. But, The Outcomes Team found the advice hard to follow: the more the questions were able to capture depth of need, the more likely they were to deal with sensitive, upsetting, confusing, or private topics (and so, be rejected by service users with some of the most acute needs). The easier questionnaires were to complete (and so, the more likely to include a fuller cohort of service users), the more likely they were to skim over or avoid the challenges in people’s lives. The most challenging problems would be excluded either way, through data performativity (Blouin 2020). Some people, and some problems, simply could not bear authentic measurement within available forms.

For example, (a fictionalised case): 17-year-old Colette had been permanently excluded from three different mainstream schools. She was becoming increasingly estranged from her adoptive mother, who she described as ‘cold’ and ‘cruel’. Her younger birth sister, who was still living with her birth mother, had recently reached out via social media, after years without contact, and Colette was hoping to take over parental care of her sister as soon as she turned 18. The only problem was that social services were unlikely to trust her with her sister’s care, given that her relationships with much older men had led to Colette being placed on a ‘Child in Need Plan’ some years earlier, to address a lack of safeguarding in her adoptive home. Colette didn’t think social services would let her care for her sister if she maintained her current relationship with a much older man, since he may pose a risk to her sister, but she also hated that her adoptive mother used the Child in Need plan as a reason to take excessive, even, "vindictive" (from Colette’s perspective) control over her life. What box should she tick, on a 5 point scale, when asked to rate: "I have been feeling close to other people"? And, is it fair to ask her that question?

Colette completed all of the items on her first questionnaire except the ‘close to others’ question, which she left blank. At end of her second 12-week programme, so, on her fourth time presented with the same measure, she did finally give a score to that question (‘3’)– but without a ‘T1’ measure to act as the ‘before’, Colette’s capacity to eventually record a response was rendered statistically irrelevant.

Anthropologist Niel Armstrong uses the term ‘routinized intimacy’ for the asymmetrical requirements that audit measures place on NHS mental health service users to bare all, efficiently and unreservedly, as part of a procedural occurrence like form-filling or symptom reporting (2016, pp. 205, 236, 2023, p. 7, see also, Bruun 2023). While he notes that there are some positive aspects of routinized intimacy for both patients and clinicians (quantifying a problem can make it more emotionally approachable), overall, he assesses routinized intimacy as posing risks to the development of caring relationships by dint of the extractive, standardised manner in which sensitive topics are recorded. Similarly, I felt the routinized intimacy involved in outcome measurement was not only an unfair to ask of many young people, it was also an unrealistic expectation—those with emotionally complex predicaments or without the required emotional literacy/numeracy were unlikely to be *able* to do it accurately or authentically, even if they were willing, and being presented with the request, risked loss of trust in those providing care.

### Data roles

Here, it is useful to introduce the idea that young people are expected to fulfil *a role* within the care-measurement assemblage. Sonja Erikainen and colleagues use the term ‘role’ to describe how infrastructural shifts in healthcare made possible by Big Data risk merging the previously distinctive social and moral positions of patient, consumer, and research participant (Erikainen et al. 2019). Klaus Hoeyer uses the term ‘roles’ (2019, and references Parsons 1951) to show how policies of personalised medicine place responsibility on individuals to track their health and engage in preventative measures, all while subscribing to the (as yet, undelivered) promise that health data will yield better, fairer public health at some point in the future. In contrast (but in support of those arguments), I intend the term ‘data roles’ to bring with it a stronger resemblance to Parsons’ interest in the *legitimacy* of health claims (1951, 1975 not withstanding the substantial and valid critiques of that term, beyond the scope of this paper to collate, Burnham 2012, 2014; Fahy and Smith 1999).

Parsons argued that society accommodates those who are incapacitated by legitimate sickness, by not expecting usual activities of them (he notes school and office work as prime examples). In return, society expects those who are ill to subscribe to medical advice and get themselves better as soon as they are able, cases of malingering are much maligned, so that overall, trust endures. A key actor in the maintenance of trust (and social control^5^) is the physician, who personally and professionally carries authority to legitimise illness and to support the sick to play their own part in actively trying to get well. Through Parsons’ functionalist account, the system can look like a magnificent, delicate, balance between self-interest and collective cohesion (Frank 2013). While it is important to recognise that a *rhetoric* of functionalism persists empirically today (e.g. in the notion that data can lead to ‘healthy’ moral economy of public spending, Hoeyer 2019; Davis et al. 2023, p. 457), Parson’s analytical functionalism is not helpful to my project. In contrast, current use of the term ‘role’ (as in the examples above) benefits from the critical associations that the term now instantly evokes, thanks to Marxist and feminist turns since Parson coined his phrase. The term ‘role’ comes already clustered by critical questioning: 'role in what?' 'for whom?' 'to what ends?'

Yet, while the functionalism of Parsons’ account has been long surpassed, subsequent theoretical apparatus for observing the means of accruing legitimacy in health care often retain a strong focus on the structuring impact and agentive possibilities of *human-held* cultural expectations about how needs should present (e.g. in theories of interactionism, performativity, narrative, or patienthood). What becomes clear, in relation to the care-measurement assemblage described here, is that accruing worth and legitimacy is not merely a sociological process, but instead, a socio-*technical* one. Incorporating STS-led observations of the power of things within systems of health and care, the term ‘data roles’ can enliven the critical point that people may not only be *expected* to fulfil new sorts of measurement roles in the era of digitalised health, but that their capacity to make legitimate claims on society for support, care, or accommodation, may *rely upon* those data roles being adequately fulfilled. A final clear departure from Parsons is necessary: it is more helpful to think of these data roles as pertaining to *needs*, rather than ‘sickness’, to incorporate more varied phenomena as well as to embrace non-pathologising language. We all have needs, some of which must be counted in order to count.

Young people could defy the data roles expected of them. But as the final section of this case study moves to observations of such defiance, this serves to illustrate that embodied amenability is part of the role young people are expected—but not obliged—to fulfil.

### Embodied amenability

The first group to fill out the new Outcome Measure at The Stables were from a Pupil Referral Unit, Meadow College, who provided education for young people who had been excluded from mainstream schools. Meadow College had been referring students to The Stables for 6 years previously, sometimes in small groups and sometimes as individuals for private sessions. Meadow College had a group of eight enrolled onto a 12-week programme. The plan was for two different outcome measures to be filled in during sessions two and eleven (since session one prioritised introductions and relationship building, and session twelve was thought potentially trickier as a transition out of the intervention).

The completion of measures was not at all straightforward.

On the morning of session two, a group of six young people arrived on a minibus, accompanied by three teachers. Two young people were absent. Visits to The Stables tended to have less absences than normal school days, but it was rare to have a full roster of enrolled students. The minibus was twenty minutes late, it had waited for a late student to arrive. There had been an argument on the bus. One of the young people, River, launched off the bus first, at speed, and began pacing in the corner of the carpark, occasionally darting back towards the cohort exiting the bus to shout insults, while holding back tears. Two of the others, Jayden and Charlie, seemed exhilarated by the outing to The Stables and began approaching horses before the session was underway. When tactfully redirected by Stables staff one stole the other’s coat. The teachers were anxious to get going with the activities as quickly as possible, they saw the outcome measure as a favour to The Stables. Another young person, Derek, swayed from foot to foot, standing near one of the teachers, nervously wringing his hands. He had a tick—a regular, almost persistent, twist of his neck and lift of his right elbow, which was particularly pronounced amid the drama of the arrival, and which was likely to make handwriting the questionnaire challenging. One teacher attempted to coax the final young person, Freya, off the bus. I could just about see across the carpark, through the window, that she was sat on the back seat with her eyes fixed downward and jaw clenched—she would stay in that position for the next thirty minutes, not responding to efforts to draw her into dialogue or engagement. She would later join the session, silently, watching from the back, rather than partaking actively, and her teachers would remark that this was a great success given her usual complete withdrawal from all events and activities.

Skilfully, the session leader, Jenny, gathered as many young people as possible around picnic benches in a quiet spot. Jenny was supported by two further members of The Stables staff, the PRU teachers, and me as volunteer. Jenny explained there was some paperwork to complete before the equestrian activities begun. It was the SWEMBWS T1 measure. A clipboard was fetched for River, since she refused to sit with the other students. I asked if I should take a clipboard to the minibus for Freya, but Jenny thought that was a bad idea, she’d have to fill her form in another time, or perhaps, it was best not to get her to complete it at all—the thin line between her involvement and non-involvement was too precarious to test. Charlie at one point stood on the seat with both feet, and reached down to fill in the form at arms’ length, holding the very end of his pen in his finger tips. He ticked all 4s and 5s on the five-point scales, though the ticks were oversized and barely within the boxes, so some interpretation was required, and he hadn’t put his name on the top of the sheet. When gathering the forms, Jenny mused it may have been better to do the forms the following week when Charlie was more settled. She felt his high T1 scores were inaccurate and risked distorting the fragile overall statistical evidence of changes brought about by The Stables.

No matter what items feature within psychometric measures, PROMs presume respondents are willing and able to direct their embodied attention towards the measurement task at an opportune moment. Some aspects of some young people’s behaviours were reminiscent of Paul Willis’ classic descriptions of working class ‘lads’ refusals to subscribe to the embodied control inherent within the schooling system (1978). Willis argued that for lads who had learned to value control over, and with, their own body, the offer to give up control over ones’ movements in exchange for scholarly knowledge was simply not a good bargain.

Reviving Willis is helpful in drawing attention to the too-easily invisible expectations of embodied acquiescence that accompany schooling, and, in my study, outcome measurement. But in the time since Willis’ classic work, educational sociologists’ understandings of neurodiversities, somatic aspects of trauma, and the affective and psychosocial aspects of habitus have broadened understandings of why it might be that some young people do not act in a way that is somatically conducive to dominant pedagogical expectations (e.g. Bresler 2013; Ivinson 2012). At The Stables, young people’s bodies were not amenable to pre-determined, predictable, authority-led direction in a range of ways, above and beyond the sorts of strategic and deliberate behaviours that Willis describes. This included fidgeting and pacing, shutting down with hands wrapped around the torso and attention on the floor, shaking with rage or frustration, feeling too nauseous to concentrate, freezing with worry, an embodied limpness that looked like apathy, and many more variations of not being ready, willing and able to concentrate on something like ‘the paperwork’ of outcome measurement.

This lack of embodied amenability should be considered in a broader sense than deliberate compliance or resistance, including, for example, those who may not be in the right place at the right time for a range of circumstantial reasons associated with the precarities of life (lack of transport from a new address, crippling anxiety which meant they couldn’t leave home, an urgent family issue arising, a late taxi driver, a conflict with a school teacher leading to refusal to attend etc.), meaning that measurements were missed, rushed, or delivered at an inopportune time. Some of the work The Stables’ staff were most proud of took place in the time before the first measure was feasible (which was sometimes, not until week 5, 6, or 7), in bringing young people around to a state in which they were amenable to measurement. This is work that sits below, or before, the baseline—or, to put it another way, the baseline required a more measurable target population than was accurate or realistic (as Ryder, Edwards and Clements describe for care-experienced young people also, 2017:, p. 6).

One of the youth workers at The Stables, Robin, made the astute observation that it seemed much easier for session leaders to draw young people, whether in individual sessions or group sessions, into tasks like clearing up after horses, making feeds, brushing horses, walking with horses, and so on, when compared to the sense of pulling teeth that sometimes accompanied any written task, outcome evaluation just one among them. Such written tasks included (variably, at different services) learning journals, session reflections, goal setting sessions, or group work tasks like planning a session for the following week. Some equine-assisted programmes specifically aimed to re-introduce written, classroom-type work as part of the intervention, using horses as the more palatable focus (e.g. calculating correct weights of hay given the size of the horse, in order to ‘phase in’ mathematics). While these tactics were often successful, it was clear that tactics were needed, since paperwork seemed physically repellent to some of the young attendees. Some held paper sheets at arm’s length, tried not to look at them, or sit near them, doodled across them, dropped them into the mud, and either rushed and/or delayed completion. Some of The Stables’ cohorts may have had substantial, persistent experience of school-like tasks that carried unreasonable and unachievable expectations, and that made them feel unwelcome, inadequate, substandard, or like a problem that needed solving (McDermott and Varenne 2006; Varenne and McDermott 2018). Young people’s evaluative responses to health measurements (or, what Fiore-Gartland and Neff call ‘data valence’ (2015)) was entwined with their evaluative responses to schooling. Robin’s observation is critically important, because it infers that it is the young people already most disenfranchised by schooling that are likely to be further marginalised by these encounters with the care-measurement assemblage.

### Pushing/supporting measures

The Stables staff didn’t withhold services from those who couldn’t or wouldn’t complete measures (as in the case of Freya above), but they were also impressive in their use of tact and innovation in order to get measures completed without too substantively disrupting sessions or relationships with service users. This puts mental health outcome measurement within the category that Deborah Lupton calls “pushed self-tracking”, meaning “Self-monitoring…taken up more or less voluntarily, but in response to external encouragement or advocating rather than as a wholly self-generated and private initiative “(Lupton 2016, p. 107). Some staff had conscientiously experimented with different timings (at the start, middle, or end of a session) and different locations (in the paddock, arena, classroom, or picnic benches; in privacy or in company). One service even asked its staff and volunteers to deliberate: should the dogs be allowed out during outcome measurements to soften the mood, or put away, to prevent distractions? These experiments seemed to produce mixed, rather than clear, results.

Staff from different services talked to me about their use of body language, and verbal language, to set a tone that would make the measures feasible. James told me that generally a positive and pragmatic ‘let’s just get this done then we can get on with the session’ approach worked best; while Corin felt that it was important to be gentle, empathic, and always ready for questionnaires to throw up some unexpected responses. Early into her professional career, in a more conventional office-based therapy setting, one of her clients had once written "I want to die" across a psychometric measure and then run off, somehow gained access to the staff break room, and barricaded themselves in it. The experience had left her understandably anxious when collecting measures, and frustrated at the need to do any sort of paperwork that rushed or obscured the building of careful, trusting relationships. Many service providers talked about helping young people to complete the measures, through explaining difficult terms in the questionnaire wording (e.g. ‘optimistic’), redirecting attention towards the measurement task, reading out questions, acting as a scribe, or helping young people work through their reasoning for giving certain scores. Sometimes, session leaders gently explicitly evoked the sense of role, or duty, in getting the measures done—particularly if a young person asked why they had to complete the measure. I heard those questions answered in these ways: “Because people will want to see whether what we do for you is making a difference”, or “it’s just something that funders need to be completed in order to pay for you, and other kids, to come here”.

While service leaders tried to buffer service users from the uncomfortable requirements of measurement, by selecting as best tools as possible, and implementing them as flexibly and tactically as they could (and sometimes, not at all), service leaders also hoped that enough young people could and would fulfil the role of providing ‘good’ outcome measures in order to demonstrate their value in legitimated terms. “In the long run”, clinical psychologist and Outcomes Team member Peter told me, perhaps reassuring himself: “completing outcome measures *is* in their best interests… we need to make sure the services they are offered are working for them, and we need to give them a say in that”.

## Discussion: “Impossible situations” of “legitimacy capture”?

Political theorist Bernardo Zacka describes ‘impossible situations’ as those faced by street level public service personnel, grappling with requirements to measure their performance in ways that don’t represent the moral values that they associate with their work (2017). Zacka argues that impossible situations ought not be brushed over as a matter of personal ethics and strategy, for personnel to figure out as though each is faced with the pragmatic problem of round pegs and square holes. Rather, such situations ought to be taken as indicative of deeper tensions between competing aspirations for public policies.

Service providers at The Stables and beyond may all have recognised challenges in collecting good data, but they, like Zacka’s bureaucrats, were liable to tackle these challenges personally and strategically, as largely unavoidable, unpolitical, pragmatic issues that had to be dealt with in a best foot forward, ‘don’t let the perfect be the enemy of the good’ sort of manner, because, as Peter attested, *not* collecting outcome data seemed unthinkable. When they asked for "help with data" in my opening vignette, service providers wanted round peg-square hole strategies, not a political campaign. Yet, through analysing the data roles expected of young people in accruing legitimacy for the services that support them, this article shows that it isn’t only service leaders who face unresolvable challenges when it comes to getting measures done, it is also service users who are put in an impossible and unfair predicament. This warrants political critique above and beyond personal strategy.

The concept of data roles supports such critical questioning along two parameters:

What do these data roles ask of people?

And;

What do these data roles do for people?

The young people at The Stables were asked to fulfil a challenging data role in relation to their mental health care, involving embodied amenability, emotional literacy/numeracy, routinized intimacy, the ‘owning’ of fixable problems and the subscription to particular, standardised, normative ideals about healthy subjectivity. Young people did have the political affordance not to acquiesce with these roles. They could fill in measures in unexpected ways, (missing out items, not paying attention to them so that the data is not authentic, rejecting routinized intimacy by ticking all ‘5’ etc.), and they could simply not fill them in at all. These instances shouldn’t be taken always as deliberate acts of resistance against measurement, so much as an indication that the data roles ask more of young people than they are able or willing to afford. Freya, for example, wasn’t deliberately critiquing the outcomes measure when she wouldn’t leave the minibus, she had other stuff going on that rendered her needs unmeasurable in the given format. The data role assigned to young people expects them to be more measurable than Freya (or Colette, or Charlie).

And what do these data roles do for young people, systemically? While the explicit aim is the quality assurance of diverse forms of treatment and care, the risk is that young people are co-opted into building a data set that measures away their own diversity, complexity and context-dependency. But if young people defy their data roles through not measuring, or not measuring progress, then the most complex predicaments, the most marginalised diversities, and the most flexible, inclusive services, are at risk of further precarity in a competitive system that prizes evidenced fixability. Nguyen calls it ‘value capture’ when people defer to data to evaluate their lives (2024), but in the case presented here, such deferral is not just a personal temptation (on the part of service users or service providers), but a compelling requirement of the way care is organised, legitimised, paid for and delivered within a care-measurement assemblage, leaving young people precarious in terms of public recognition of their needs and predicaments, either way (whether they do, or don’t complete these forms).

In sum, the data roles currently expected of young people in alternative mental health care ask too much of them, and do too little for them.

Yet while their defiance of these roles produces precarities, there are some grounds for optimism. Thus far the data simply doesn't yeild to the pressure to produce competitive stories of fixability—thanks, largely, to the integrity of service providers, who, somewhat remarkably, do not tend to manipulate or invent their numbers. As yet, in the case presented here at least, data roles are not fully realised, care is provided precariously on the margins of the care-measurement assemblage, outscoping or defying the data-led mechanisms through which the public legitimacy of needs and responses is supposedly configured. At present, this is a counter-example of Rose’s governmentality—young people defy measurement, and do not subscribe to the forms of self-monitoring on offer, making the problem of youth mental health hard to politically manage (and certainly, hard to manage in such a way that the legitimacy of authorities is bolstered when measures are persistently missing).

If enough young people continue to defy their data roles on the terms currently available, will more appropriate, inclusive, empowering forms legitimacy gain traction for data marginalised people and the services that support them? Following Zacka, solutions to such unmeasurability may well require remediating unresolved inconsistencies between portrayals of the nature of the problem (e.g. where the problem is located, who ought to put it right, and what ‘better’ would look like). Following Hoeyer, resolving unmeasurability might involve a public recognition of the complexity of needs in the present, over and above deferring to the hopes for some data-led version of fairness and efficiency to be found in the future (2019). Following Armstrong (2023) and Nguyen (2024), appropriate evaluation may need to rely more on the subjective, morally driven, personally varied evaluations of need, worth and care, rather than the scalable reductions of data. Will any of these, or other, options shift, or challenge, the current momentum of the care-measurement assemblage, or will the most complex needs, and the most flexible, inclusive services, be pushed into further precarity? Thinking with data roles will help us to stay critically attuned to how legitimacy is being configured, not through the authority of any particular person (doctors, politicians), nor through the collective agreement of the social body (through function, or discourse), but through care-measurement assemblages that may not reflect the values or needs of the people contributing to them.

## Data Availability

No raw data is available for sharing due to the identifiable and sensitive nature of the data.

## Footnotes

1 via NHS commissioning; referrals from the Children and Adolescents Mental Health Service; or Local Authorities’ spending on Education and Health Care Plans.

2 I prefer ‘unmeasurability’ to the more grammatically correct ‘immeasurability’. The latter suggests wondrous, unfathomable excess, whereas the former rhetorically references ‘the unspeakable’ and ‘the untouchables’: conditions of exclusion and judgement. Unmeasurability suggests that somebody, or something, has been rendered outside of (perhaps even ‘below’) recognition.

3 Data/Audit within Health Service Framework in relation to children’s mental health: DH 2004:168–169.

4 Department of Health 2011, Department of Health 2015: 57–62, The Mental Health Task Force 2016: 49–54, NHS 2019: 111, NHS 2020:11–13.

5 Parson’s account has more critical potential than it is often credited with, for example, he recognises a non-essentialising notion of ‘illness’, as ‘societally legitimised incapacity’ (1975), which leaves space for critique regarding inappropriate societal expectations for the ‘proper’ capacity of its members.
